# Serum TNFα and IL-17A levels may predict increased depressive symptoms: findings from the Shika Study cohort project in Japan

**DOI:** 10.1186/s13030-024-00317-5

**Published:** 2024-10-02

**Authors:** Hirohito Tsuboi, Hiroyuki Sakakibara, Yuuki Minamida-Urata, Hiromasa Tsujiguchi, Akinori Hara, Keita Suzuki, Sakae Miyagi, Masaharu Nakamura, Chie Takazawa, Takayuki Kannon, Jiaye Zhao, Yukari Shimizu, Aki Shibata, Aya Ogawa, Fumihiko Suzuki, Yasuhiro Kambayashi, Tadashi Konoshita, Atsushi Tajima, Hiroyuki Nakamura

**Affiliations:** 1 Graduate School of Human Sciences, The University of Shiga Prefecture, 2500 Hassaka-Cho, Hikone, 522-8533 Japan; 2 Research Group of Psychosomatic Medicine, Faculty of Pharmacy, Pharmaceutical and Health Sciences, Kanazawa University, 1 Kakuma-Machi, Kanazawa, 920-1192 Japan; 3 Department of Hygiene and Public Health, Faculty of Medicine, Institute of Medical, Pharmaceutical and Health Sciences, Kanazawa University, 13-1 Takara-Machi, Kanazawa, 920-8641 Japan; 4 Graduate School of Agricultural Science, Kobe University, 1-1 Rokkodai, Nada-ku, Kobe, 657-8501 Japan; 5 Department of Bioinformatics and Genomics, Graduate School of Advanced Preventive Medical Sciences, Kanazawa University, 13-1 Takara-Machi, Kanazawa, 920-8640 Japan; 6 Innovative Clinical Research Center, Kanazawa University, 13-1 Takara-Machi, Kanazawa, Japan; 7 Department of Biomedical Data Science, School of Medicine, Fujita Health University, 1-98 Dengakugakubo, Kutsukake-Cho, Toyoake, 470-1192 Japan; 8 Department of Nursing, Faculty of Health Sciences, Komatsu University, 10-10 Doihara-Machi, Komatsu, 923-0921 Japan; 9 Department of Geriatric Dentistry, Ohu University School of Dentistry, 31-1 Misumido, Koriyama, 963-8611 Japan; 10 Department of Public Health, Faculty of Veterinary Medicine, Okayama University of Science, 1-3 Ikoinooka, Imabari, 794-8555 Japan; 11 Division of Diabetes Endocrinology and Metabolism, Yachiyo Medical Center, Tokyo Women’s Medical University, 477-96 Owada-Shinden, Yachiyo, Japan

**Keywords:** Low-grade inflammation, Depressive symptoms, Inflammatory cytokines, Interleukin (IL)-17, Tumour necrosis factor (TNF)α, Community-based cohort study

## Abstract

**Background:**

Low-grade systemic inflammation may be a key player in the immune activation that has been reported for mental health deterioration. We hypothesised that elevated serum levels of inflammatory cytokines increase neuroinflammation and exacerbate depressive symptoms.

**Methods:**

The participants were part of a cohort study for whom data was available for both 2015 and 2019. In 2015, blood samples were collected from 232 participants. Their depressive symptoms were assessed both 2015 and 2019 using the Centre for Epidemiologic Studies Depression Scale (CES-D) (*n* = 33). The multiplex immunoassay system (Luminex® 200) was used to measure the serum concentrations of IL-6, IL-10, IL-12, IL-17A and TNFα. Data were analysed using linear models with the level of significance considered to be *p* < 0.05.

**Results:**

After controlling for age, BMI, smoking and alcohol consumption, in 2015 the serum concentrations of IL-17A and TNFα in 2015 were significantly positively associated with the CES-D scores of women (standardised *β* (*B*) = .027, *p* < 0.01 and *B* = 0.26, *p* < 0.01, respectively). The serum concentrations of IL-17A and TNFα of men were significantly positively associated with the CES-D scores of 2019 (*B* = 0.62, *p* = 0.02 and *B* = 0.59, *p* = 0.02, respectively).

**Conclusions:**

In this cross-sectional study, we found a significant positive correlation between the depressive symptoms and serum TNFα and IL-17A levels of women. In addition, our longitudinal findings suggest the possibility that TNFα and IL-17A could elevate the depressive symptoms of men.

## Introduction

Theories on serotonergic dysfunction and cortisol hypersecretion do not provide sufficient explanations for the nature of depression or depressive symptoms. There is now evidence that there are pathways and mechanisms by which the immune system can influence the brain and behaviour. The central nervous system (CNS) mainly affects the immune system via the neuroendocrine outflow and the autonomic nervous system. Conversely, the immune system, including inflammation, modulates the CNS. Chronic inflammation differs from normal or acute inflammation in that the body is unable to suppress the immune response, which thus results in continuous systemic low-grade inflammation. A link between inflammatory indicators and psychological or psychiatric conditions has been reported in otherwise healthy individuals with depressive symptoms and in those with clinical depression. For example, low-grade systemic inflammation has been examined in relation to clinical depression and/or depressive symptoms [1, 2]. Previous studies have shown that chronic inflammation in various peripheral organs can render the brain vulnerable [3, 4]. The authors of some review articles have suggested that the mechanism of neuroinflammation and neurodegeneration in the CNS may be linked to peripheral inflammatory signalling [5, 6]. In the clinical field, a meta-analysis of studies into major depressive disorder (MDD) concluded that two pro-inflammatory cytokines, interleukin (IL)-6 and tumour necrosis factor-alpha (TNFα), were consistently elevated in depression, while other cytokines, such as IL-1β, IL-4, IL-2, IL-8, IL-10 and interferon-gamma (INFγ), were not [7]. Other meta-analyses have suggested associations between depression and elevated levels of inflammatory markers, such as C-reactive protein (CRP), IL-6 and, to a lesser extent, IL-1 [8, 9]. While psychological stress can lead to depression and peripheral inflammation efferently through the hypothalamic–pituitary–adrenal (HPA) axis or the sympathetic pathway, peripheral inflammatory signals can activate an inflammatory response in the CNS afferently via the blood–brain barrier (BBB) or the vagus nerve and cause depression or other disorders [10]. However, the effect of peripheral inflammation on the CNS is poorly understood, although there is increasing evidence implicating neuroinflammation in the pathogenesis of depressive disorders [10, 11].

The pro- and anti-inflammatory cytokines, TNFα, IL-6, IL-17A, IL-12 and IL-10, were selected for our investigation of the effects of chronic inflammation on the CNS. TNFα is a cytokine and an adipokine that promotes insulin resistance and activates inflammatory pathways [12]. It is derived from adipose tissue and accelerates low-grade inflammation by establishing a vicious cycle between adipocytes and macrophages [13]. For this reason, measuring TNFα is essential for evaluating the health of modern people. IL-6 acts as both a pro-inflammatory cytokine and an anti-inflammatory myokine [14]. It is a multifunctional cytokine produced by many types of cells, including immune cells, endothelial cells, fibroblasts, myocytes and adipocytes, mediating inflammatory as well as stress-induced responses [15]. In a previous study, we found a positive association between depressive symptoms and peripheral IL-6 concentration [16].　IL-12 is the major Th1-polarising factor that enhances inflammation by activating macrophages [17]. Contrarily, it can induce neuroprotective tissue adaption that prevents early neurodegeneration and sustains trophic factor release during neuroinflammation [18]. IL-12 represents an ideal candidate for neuroinflammation or neuroprotection. IL-17A is a pro-inflammatory cytokine that is mainly secreted by T-helper 17 (Th17) lymphocytes but also by other T cells, granulocytes, monocytes and natural killer (NK) cells [19]. It is involved in atopic allergic inflammation including asthma, allergic rhinitis and conjunctivitis, atopic eczema, and food allergies [20]. The relation of atopic and allergic diseases to anxiety or depression has often been reported in the relation. One reason may be due to IL-17 increasing BBB permeability in a dose-dependent manner [21]. In addition, we reported elevated levels of serum IL-17A and higher depressive symptoms [22]. IL-10 is an anti-inflammatory cytokine produced by Th2 cells that has multiple pleiotropic effects in immunoregulation and inflammation. It downregulates the expression of Th1 cytokines and can block NF-κB activity that mediates inflammation, including IL-6 production and Th17 differentiation and its responses [23].

Despite the increasing reports of associations between peripheral chronic inflammation and depression, there is a paucity of studies involving members of the general population. Although they are closely related, it is important to distinguish between clinical depression and depressive symptoms in daily life. Preventive approaches to depression have been shown to significantly reduce the incidence of MDD [7]. This study was done to clarify the association between serum levels of inflammatory indicators and depressive symptoms among a cohort of participants from the general population, by identifying the relation between the levels of peripheral cytokines and the depressive symptoms of members of the community.

## Methods

### Study population

The participants were part of the Shika Study cohort project for whom data was available for both 2015 and 2019; this project has been carried out in the Noto Peninsula, Ishikawa, Japan, since 2011. The project aims to identify solutions to address lifestyle diseases by investigating community members aged 40 years or more. Details of this study have previously been reported elsewhere [22]. All of the participants were literate, understood the Japanese language well and were requested not to use proxies. The study protocol was approved by the Ethics Committee at Kanazawa University (on 18 December 2013, receipt number 1491). Written informed consent was obtained from all participants.

### Procedures

**Fig. 1:**
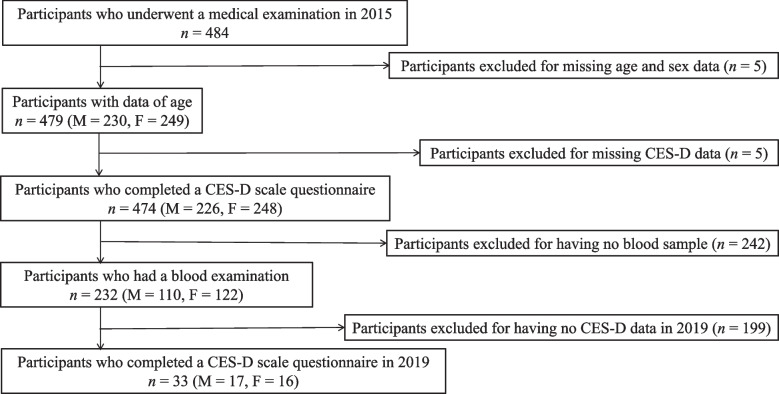
Flow chart showing the participant recruitment procedure

Blood was sampled in 2015. Demographic and questionnaire data were collected in both 2015 and 2019. Figure 1 shows the study procedure.

#### Questionnaire

A self-administered questionnaire was distributed to participants in advance, and the completed questionnaires were collected on the day of their medical examination. The entire process was conducted paying careful attention to the protection of their privacy. The questionnaire included items relating to demographic characteristics (age, sex, height, weight, present health status, diseases, medication, etc.) and lifestyle characteristics (smoking status, alcohol consumption and if they lived alone). Depressive symptoms were assessed using the Japanese version of the Centre for Epidemiologic Studies Depression Scale (CES-D) [24, 25]. Of the 492 persons initially recruited in 2015, 409 participants (200 males, mean age ± standard deviation (SD) = 59.1 ± 11.5; 208 females, mean age ± SD 61.1 ± 12.9; 1 unknown) out of 492 gave their consent to participate in this study in 2015. In 2019, the same procedure was carried out, with 96 men (mean age ± SD = 64.8 ± 10.28) and 116 women (mean age ± SD = 61.2 ± 10.70) from the original cohort consenting to participate.

#### Blood collection

Fasting blood sampling was done between 0800 and 1200, with blood taken from the forearm vein was collected in heparinised and serum-separator vacutainer tubes, after which sera was separated via centrifugation. The serum samples were delivered to Kanazawa University through a commercial laboratory (SRL Kanazawa Laboratory, Kanazawa, Japan). The sera were frozen and stored at − 30°C until the assays were performed two months after sampling.

#### Assays for inflammation-associated factors

The serum samples were tested for TNFα, IL-6, IL-10, IL-12p70 and IL-17A using a multiplex human immunoassay kit (Luminex® 200TM) with the human high sensitivity T cell panel (Merck Japan, Tokyo, Japan).

### Statistical analysis

The Japanese version of IBM SPSS Statistics 29 (IBM Japan, Tokyo, Japan) was used for data analysis. An unpaired t-test was used for comparisons between two groups, Pearson’s correlation test was used to explore correlations between two groups, following, a linear regression analysis done to adjust for confounders. The level of significance was considered to be *p* < 0.05.

## Results

Table 1 presents the participants’ characteristics. Significant differences between men and women were observed for BMI and serum IL-17A concentration, with both variables being significantly higher in men than women (t-test: *t* = 3.86, *p* < 0.0005 and *t* = 2.75, *p* = 0.006, respectively). There were no significant differences between the sexes for other variables, including smoking, alcohol consumption, and if they lived alone.

**Table 1:** Demographic and clinical characteristics of participants

	Men				Women				Differences	
	*N*	Mean	S.D	Range	*N*	Mean	S.D	Range	*t* value	*p* value
Age (years)	111	59.95	11.754	40–87	122	59.78	12.502	40–84	0.11	0.92
BMI (kg/m^2^)	111	24.354	3.0722	17.6–32.7	121	22.64	3.6453	16.1–32.9	3.86	< 0.0005
CES-D score (2015)	111	10.2	7.456	0–36	122	10.56	8.218	0–44	0.93	0.36
CES-D score (2019)	17	11.41	8.938	0–33	16	8.75	7.28	0–25	0.35	0.73
TNFα (pg/mL)	110	5.82	2.191	1.75–14.75	122	6.19	2.298	1.46–16.27	1.25	0.21
IL-6 (pg/mL)	110	3.43	3.041	0.00–24.18	121	3.3	3.194	0.00–32.98	0.31	0.76
IL-10 (pg/mL)	110	13.79	19.499	0.00–152.21	122	14.73	12.487	0.00–72.51	0.44	0.66
IL-12 (pg/mL)	110	3.51	2.276	0.20–10.18	121	3.92	2.28	0.00–13.09	1.38	0.17
IL-17A (pg/mL)	110	5.33	4.032	0.00–20.43	122	7.34	6.864	0.00–35.78	2.75	0.01
	*N*				*N*				*χ*^*2*^ value	*p* value
Smoking habit										
Non-smoker	22				95				79.7	< 0.0005
Ex-smoker	53				12					
Current smoker	36				15					
Alcohol consumption										
Yes	73				35				13.8	< 0.0005
No	38				87					
Living alone										
Yes	8				7				0.21	0.65
No	101				111					

Continuous variables were examined using unpaired t-tests and categorical variables using chi-square tests

*BMI* Body mass index, *CES-D* the Centre for Epidemiologic Studies Depression Scale, *IL* Interleukin, *TNF* Tumour necrosis factor

**Fig. 2:**
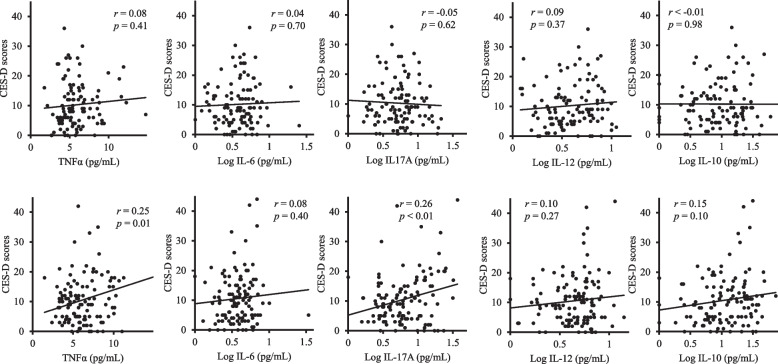
Correlations between depressive symptoms and serum cytokine concentrations in 2015. In 2015, significant positive correlations between CES-D scores and serum concentrations of the cytokines TNFα and IL-17A were observed in women. IL-6, IL-10, IL-12 and IL-17 concentrations were base 10 log-transformed. CES-D: the Centre for Epidemiologic Studies Depression Scale

**Fig. 3:**
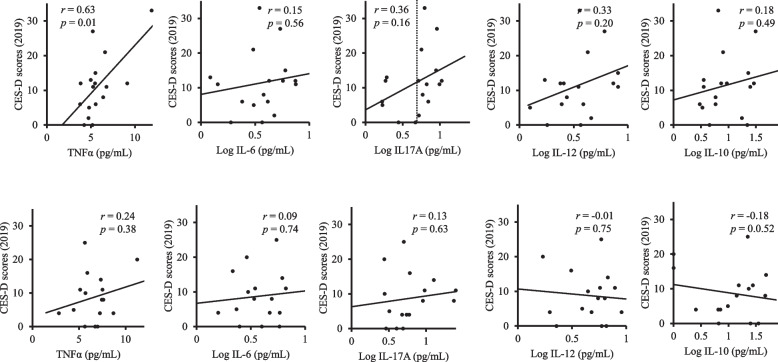
Correlations between depressive symptoms in 2019 and serum cytokine concentrations in 2015. In 2019, a significant, positive correlation between the CES-D scores and serum concentrations of the cytokine TNFα was observed in men. As to the CES-D scores of men in 2019, when IL-17A concentrations were divided into higher and lower groups with a cutoff at 3.35 pg/mL, the serum IL-17A level of the higher group showed significantly increased CES-D scores in 2019 in comparison with that of the lower IL-17A group (t-test: *t* = 2.63, *p* = 0.019). IL-6, IL-10, IL-12 and IL-17 concentrations were base 10 log-transformed. CES-D: the Centre for Epidemiologic Studies Depression Scale

In 2015, significant positive correlations were observed for women between the CES-D scores and the concentrations of the serum cytokines TNFα and IL-17A were observed among women (simple correlation: *r* = 0.25, *p* = 0.006 and *r* = 0.26, *p* = 0.003, respectively) (Fig. 2). In 2019, a significant positive correlation was observed for men between the CES-D scores and the concentration of the serum cytokine TNFα (simple correlation: *r* = 0.63, *p* = 0.007) (Fig. 3). There were no significant correlations between the CES-D score and the serum concentration of any of the other cytokines in 2015 or 2019. For the CES-D scores in 2019, when the cutoff for IL-17A concentration was set at 3.35 pg/mL, the serum IL-17A level of the higher group (mean ± S.D.: 16.0 ± 59.12, *n* = 9) showed significantly higher CES-D scores in 2019 in comparison with those of the lower IL-17A group (6.3 ± 9.12, *n* = 8) (t-test: *t* = 2.63, *p* = 0.019). After adjusting for confounders such as age, BMI, smoking, alcohol consumption, and if the participant lived alone, regression analyses were performed to explore if there were links between depressive symptoms and each subject cytokine. As shown in Table 2, in 2015, IL-17A was a significant, positively related factor that could explain the CES-D scores of women in all of the models (Model 1: *F* = 8.82 (1, 109), standardised *β* (*B*) = 0.26, *p* = 0.004; Model 2: *F* = 3.63 (3, 117), *B* = 0.27, *p* = 0.003; Model 3:* F* = 2.28 (5, 115), *B* = 0.27, *p* = 0.003; Model 4: *F* = 1.85 (6, 112), *B* = 0.27, *p* = 0.003). TNFα was a significant, positively related factor in 2015 that could explain the CES-D scores of women in all of the models (Model 1: *F* = 7.67 (1, 109), *B* = 0.25, *p* = 0.007; Model 2: *F* = 3.20 (3, 117), *B* = 0.26, *p* = 0.005; Model 3: *F* = 2.00 (5, 115), *B* = 0.26, *p* = 0.006; Model 4: *F* = 1.62 (6, 112), *B* = 0.26, *p* = 0.007) (Table 2). Table 3 shows that IL-17A in 2019 was a significant, positive factor for predicting the CES-D scores of men in 2019 (Model 1: *F* = 6.90 (1, 15), *B* = 0.56, *p* = 0.019; Model 2: *F* = 2.50 (3, 13), *B* = 0.57, *p* = 0.023; Model 3:* F* = 2.15 (5, 11), *B* = 0.62, *p* = 0.017). TNFα was also a significant, positive factor for predicting the CES-D scores of men in 2019 (Model 1: *F* = 9.69 (1, 15), *B* = 0.63, *p* = 0.007; Model 2: *F* = 3.12 (3, 13), *B* = 0.61, *p* = 0.013; Model 3: *F* = 1.90 (5, 11), *B* = 0.59, *p* = 0.024) (Table 3).

**Table 2:** Linear models that explain depressive symptoms in 2015

Models		Model 1		Model 2		Model 3		Model 4	
		*B*	*p* value	*B*	*p* value	*B*	*p* value	*B*	*p* value
Men									
	IL-17A	-0.05	0.619	-0.05	0.635	-0.04	0.696	-0.04	0.674
	TNFα	0.08	0.405	0.08	0.386	0.08	0.401	0.08	0.411
	IL-6	0.04	0.697	0.04	0.695	0.04	0.663	0.04	0.670
	IL-10	0.00	0.977	0.00	0.982	0.01	0.910	-0.09	0.382
	IL-12	0.09	0.372	0.09	0.358	0.10	0.307	0.10	0.322
Women									
	IL-17A	0.26	0.004	0.27	0.003	0.27	0.003	0.27	0.003
	TNFα	0.25	0.007	0.26	0.005	0.26	0.006	0.26	0.007
	IL-6	0.08	0.400	0.10	0.288	0.10	0.296	0.10	0.306
	IL-10	0.15	0.096	0.16	0.086	0.16	0.091	0.15	0.108
	IL-12	0.10	0.272	0.13	0.184	0.13	0.177	0.14	0.145

Model 1: crude model

Model 2: Model 1 + age and BMI

Model 3: Model 2 + smoking and alcohol intake

Model 4: Model 3 + if they lived alone

Cytokine values are logarithmised with the exception of TNFα

*B*: standardised *β* value

**Table 3:** Linear models that predict depressive symptoms in 2019

Model		Model 1		Model 2		Model 3		Model 4	
		*B*	*p* value	*B*	*p* value	*B*	*p* value	*B*	*p* value
Men									
	IL-17A^a^	0.56	0.019	0.57	0.023	0.62	0.017		
	TNFα	0.63	0.007	0.61	0.013	0.59	0.024		
	IL-6	0.15	0.555	0.15	0.582	0.19	0.520		
	IL-10	0.18	0.494	0.16	0.569	0.17	0.557		
	IL-12	0.33	0.196	0.32	0.239	0.34	0.228		
		*B*	*p* value	*B*	*p* value	*B*	*p* value	*B*	*p* value
Women									
	IL-17A^a^	0.11	0.695	0.12	0.678	0.14	0.640	0.15	0.642
	TNFα	0.23	0.384	0.26	0.367	0.21	0.502	0.22	0.498
	IL-6	0.09	0.735	0.14	0.628	0.16	0.605	0.18	0.589
	IL-10	-0.18	0.518	-0.16	0.575	-0.16	0.596	-0.16	0.609
	IL-12	-0.09	0.752	-0.05	0.876	-0.03	0.921	-0.03	0.931

Model 1: crude model

Model 2: Model 1 + age and BMI

Model 3: Model 2 + smoking and alcohol intake

Model 4: Model 3 + if they lived alone

Cytokine values are logarithmised with the exception of TNFα

^a^Logistic regression analysis was performed after IL-17 levels were divided into higher and lower groups with a cutoff at 4.35 pg/mL

*B*: standardised *β* value

## Discussion

In the current study, we investigated the association between peripheral cytokine levels and the depressive symptoms of community dwellers aged 40 years and older, both cross-sectionally and longitudinally. Among women, we identified significant positive associations between depressive symptoms and their serum of TNFα and IL-17A levels. We also found that elevated TNFα and IL-17A levels were significantly associated with increased depressive symptoms among men four years after the first examination.

Depression is a complex disease with a multifactorial background [26]. Based on studies conducted to date, it is not possible to clearly define the causes and mechanisms of depression [26, 27]. However, several theories have been put forward that have attempted to explain its underlying causes, including the monoamine hypothesis, a neurotrophic theory, dysfunction of the HPA axis, and neurodegenerative and inflammatory alterations. Depression is a major public health issue and is projected to become the leading cause of disability worldwide by 2030 [28]. Although clinical depression and depressive symptoms in daily life should be distinguished, they are closely related. Preventive approaches to depression have been found to significantly reduce the incidence of MDD [28]. In the present study, we investigated the effects of low-grade systemic inflammation on depressive symptoms. Our work was informed by the neuroinflammatory theory of depression, because the inflammatory response system activates the HPA axis, leading to the production of corticotropin-releasing hormone (CRH) and adrenocorticotropic hormone (ACTH). An increase in the turnover of serotonin and catecholamines has also been reported [29].

### IL-17A

IL-17A is a pro-inflammatory cytokine that was the first member of the IL-17 family to be identified. The overproduction of IL-17A promotes hyperinflammation and tissue damage in a variety of diseases [30]. The role of IL-17 in the pathogenesis and progression of depression is a relatively new area of research that has recently been gaining emphasis. Patients with MDD have been found to exhibit increased levels of circulating cytokines, such as IL-1b, IL-6 and TNFα, which play important roles in Th17 differentiation and effector action [31, 32]. IL-17 stimulates the production of various inflammatory mediators, such as intercellular adhesion molecule 1 (ICAM-1), prostaglandin E2 (PGE2), matrix metalloproteinases (MMPs) and antimicrobial peptides, that are involved in tissue damage [33]. The induction and release of such mediators also appears to amplify IL-17 production via a positive feedback loop, thus propagating inflammatory damage [34]. Furthermore, patients with MDD who responded to treatment with antidepressants showed a reduction in plasma IL-17 levels [31]. A potential limitation of the present study is differences in the composition of fluids in the CNS and the peripheral serum, particularly because the BBB partitions the brain from circulating blood and because cytokines are relatively large molecules that cannot pass freely across the BBB. However, some studies have identified various means by which inflammatory signals may be transmitted from the periphery to the brain. Increased circulation of inflammatory factors could potentially increase the permeability of the BBB, allowing cytokines to cross, particularly through the choroid plexus and circumventricular organs [35]. Furthermore, cytokine signals could be transmitted via afferent vagus nerve fibres, triggering the release of second messengers in the brain such as prostaglandins and nitric oxide [35]. Alternatively, cytokines could cross the BBB via active transport [35]. Although little is currently known about the mechanism by which IL-17A infiltrates the brain and its mode of action, i.e. whether IL-17A acts directly in the brain or indirectly by some cascades across the BBB, some recent reports have indicated that there may be a relation between peripheral IL-17 concentration and depression. For example, patients with first-episode depressive disorder showed higher serum levels of IL-17 [36]. The IL-17 serum concentrations of patients with MDD were significantly higher than those seen in a control group [37]. Preclinical studies on animal models have shown that IL-17 induced by stress promotes depression-like behaviours [38, 39]. IL-17 is unique in that IL-17A has been reported to access the brain by disrupting the integrity of the BBB and its receptors [40], IL-17RA and IL-17RC, are richly expressed in the CNS [41]. In addition, a recent study of mice indicated that IL-17 promotes synaptic dysfunction and that neutralization of IL-17 prevents synaptic dysfunction [42]. This report also reported that anti-IL-17 treatment using monoclonal antibody appeared to be effective against such dysfunction [42].

In our present cross-sectional study of people living in the community, serum IL-17A concentrations and depressive symptoms showed a significant, positive association among women. Furthermore, our longitudinal study indicated that elevated serum IL-17A levels in men predicted higher depressive symptoms four years later. The above-mentioned mechanism of IL-17 in the CNS supports our results, although the reasons for these sex based differences are currently unclear.

### TNFα

TNFα was also shown to be related to an elevation of depressive symptoms in the current study. TNFα is a pro-inflammatory cytokine that is secreted by many types of cells and tissues. It has recently attracted attention in relation to obesity and inflammation [43, 44]. In relation to depression, it has been established that TNFα, as do IL-1 and IL-6, induces not only symptoms of physical sickness but also major depressive disorders in physically ill patients with no previous history of mental disorders [45]. In addition, studies using animal models have indicated that TNFα inhibition improved depression-like behaviour [46, 47].

The effects of TNFα on depression may be due to its activation of the HPA axis and a subsequent reduction in serotonin (5-HT) metabolism in addition to the vulnerability of the BBB to the inflammatory function of TNFα. Several findings have been made that indicate TNFα has an influence on serotonin metabolism as well as on the HPA axis. It has been suggested that TNFα activates the HPA axis during inflammatory reactions [48]. Another study suggested that TNFα and the HPA system have a mutual influence on depressed patients without inflammatory diseases [49]. Furthermore, TNFα was shown to elicit considerable decreases in 5-HT transporter function [50].

Although the precise mechanism is unknown, the present study showed associations between TNFα and the degree of depressive symptoms, cross-sectionally in women and longitudinally in men.

### IL-10

There have been contradictory findings in relation to associations between serum the IL-10 level and depression. A case–control study involving patients with MDD and individuals without depression showed that the patients with MDD exhibited higher levels of plasma IL-10 [31]. However, in the same study, patients who received antidepressant treatment exhibited elevated plasma IL-10 concentrations [31]. In a mouse model of depression, microglial but not peripheral blood IL-10 levels were reduced in learned helplessness mice; administration of IL-10 improved procognitive actions in learned helplessness mice or mice with cognitive impairments [51]. These contradictory findings in relation to IL-10 could be due to its pleiotropic effects of IL-10. It is an anti-inflammatory cytokine produced by Treg cells and plays an important role in preventing inflammation. However, it can activate Th2 cells and B cells [52] while also inhibiting to macrophages and Th1 cells and suppressing Th17 cell-mediated inflammation; thus, IL-10 can be both immunostimulatory as well as immunosuppressive.

In the current study, there were no significant associations between the serum IL-10 level and depressive symptoms.

### IL-6

Similar to TNFα, IL-6 has long been investigated in relation to the pathophysiology of depression. However, previous studies have reported contradictory findings with regard to links between elevated IL-6 levels in patients with depressive symptoms or those with MDD. This may have been due to confounders, the participants’ characteristics, or to heterogeneity among patients with MDD. For example, the IL-6 levels of patients with melancholic depression were significantly increased compared with those of healthy participants, while there was no significant difference when compared with patients with atypical depression [53]. This finding was consistent with that of another study that indicated that IL-6 levels are correlated with different subtypes of MDD [54]. A large cohort study among British civil servants (a general population or with MDD not specified) showed that higher serum levels of IL-6 at baseline were associated with subsequent cognitive symptoms of depression in both sexes at follow-up [55].

In the present study, carried out among community members aged 40 years of age or older in an urban area of Japan, there were no correlations between depressive symptoms and serum IL-6 levels, either cross-sectionally or longitudinally. However, a cross-sectional study that we carried out involving female nursing workers indicated a significant positive correlation between depressive symptoms and serum IL-6 levels [16]. The reason for these differences is unclear. It may be due to latent confounders or type-1 errors. Alternatively, it might be due to differences in the characteristics of the study cohort: the average CES-D score of participants in the present study was 10.1 in 2015, whereas in our earlier study it was 14.8 [16].

### IL-12

IL-12 is a pro-inflammatory cytokine that influences cellular immunity by promoting Th1 response. It enhances the cytotoxic activity of NK cells and facilitates cytotoxic T lymphocyte generation [56]. There are fewer reports on the relation between IL-12 and depression than there are for the other cytokines investigated in the present study. However, some studies have found elevated serum IL-12 levels in patients of MDD and rat depression models. For example, a recent study showed an increased serum IL‐12 level in patients with MDD in comparison with controls and a positive correlation between the serum IL‐12 levels and Hamilton Depression Rating Scale scores of patients with MDD [57]. Contrarily, an animal experiment revealed a protective effect of IL-12 against neuroinflammation [18]. A review article noted that the IL-12 serum/plasma level may be clinically valuable as part of a complex diagnostic approach to depression, although this must be carefully interpreted, taking into account confounders that can alter the same pro-inflammatory pathways and overlap with each other [58]. In the current study we found no significant associations between the concentration of IL-12 and depressive symptoms, possibly due to multifunctional effects of the IL-12.

### Pathways to the central nervous system

In discussion of peripheral inflammatory markers and brain reaction or depression, signals of which cytokines cross the BBB should be considered, as mentioned in the IL-17A section above. There are two types of pathways by which peripheral immune signals are transmitted to the brain, BBB-dependent and BBB-independent [59]. The BBB-independent pathways use the vagus nerve, which bypasses the BBB, whereas there are various BBB-dependent pathways, including vulnerability of the BBB itself, humoral pathways, cellular pathways including active transport, activation of NF-κB signalling and through adhesion molecules [35]. Although it was recently discovered that microglia play a role in BBB permeability during systemic inflammation [60], the precise factor remains unclear. Some inflammatory materials can evoke inflammation of the central nervous system, which can induce depression. A study of depression found that increased IL-6 levels were correlated with dysconnectivity of a brain functional network [61]. However, further research is required to clarify the pathway.

### Effects of disease and medication on immune indicators

It is essential to consider the effects of diseases and drugs on immunity. In the 2015 survey, one woman was diagnosed with rheumatoid arthritis (RA). One man and one woman had atopic dermatitis (AD). RA and AD are IL-6-related diseases that may also related to the production of other cytokines. In our statistical analysis, when we excluded the participants with RA or AD one by one, the levels of significance presented in Table 2 did not change.

It may also be necessary to exclude participants diagnosed with MDD. In our current study, we surveyed members of a community population who had not been previously hospitalised or had problems with absenteeism from work. One woman self-reported that she suffered from depression: her CES-D score was 44, the highest among the participants in 2015. The woman took an antidepressant as a sleeping aid (trazodone) and anxiolytics (alprazolam, diazepam). We found no reports regarding any relation between the above-mentioned medicines and IL-17 or TNFα [62, 63, 64, 65]. Because diazepam and alprazolam generally have contradictory effects on immune response [17], their simultaneous administration has no or very little effect on immune functions. Furthermore, the use of antidepressants to treat depression could decrease the peripheral IL-17A level [31]. In addition, this person engaged in regular physical activities during her leisure time, suggesting that her depressive status was not so severe that it affected her behaviour. Patients with MDD generally cannot continue physical activities due to loss of interest or motivation. Judging from her medication and lifestyle, the participant would not be diagnosed as having MDD: her symptoms could have been menopausal symptoms, with unidentified complaints and an accompanying sleep disorder. Her data was included this individual in our analysis. If it had been excluded, the levels of significance presented in Table 2 would have remained the same for IL-17A but not for TNFα.

### Prevention and treatment

Given that low-grade inflammation partially drives depressive symptoms, anti-inflammatory agents or non-pharmacological interventions in daily life may be beneficial. As mentioned at the last prat of the IL-17A section, treatment using monoclonal antibody may be useful [42]. A meta-analysis for anti-inflammatory treatment reported that anti-TNF antibodies (such as infliximab), non-steroidal anti-inflammatory drugs (NSAIDs) and omega-3 fatty acids improved depressive symptoms [66]. Other review suggested non-pharmacological interventions such as physical exercise, probiotics, omega-3 fatty acids [67].

### Limitations

First, a limitation of this study is the small sample size of patients evaluated using CES-D in 2019. Second, the different results by sex cannot be explained.

## Conclusions

We found that serum of IL-17A and TNFα levels were significantly, positively associated with the depressive symptoms of women, while in men they predicted depressive symptoms four years later. In the context of inflammation, IL-17A and TNFα can access the CNS and hence influence the brain neurocircuits that regulate mood, motor activity, motivation, etc. Our results provide insights that a lifestyle that protects against the generation of inflammatory responses may, at least partially, be related to preventing depression and to maintaining a healthy mental status.

## Data Availability

The authors do not have permission to share data.
